# Lateral Heterostructure Field-Effect Transistors Based on Two-Dimensional Material Stacks with Varying Thickness and Energy Filtering Source

**DOI:** 10.1021/acsnano.9b08489

**Published:** 2020-01-14

**Authors:** Enrique G. Marin, Damiano Marian, Marta Perucchini, Gianluca Fiori, Giuseppe Iannaccone

**Affiliations:** † Dipartimento di Ingegneria dell’Informazione, Università di Pisa, 56122 Pisa, Italy; ‡ Departamento Electrónica, Facultad de Ciencias, Universidad de Granada, 18071 Granada, Spain

**Keywords:** lateral heterostructures, two-dimensional materials, energy filtering source, field-effect transistor, sub-60 mV/decade subthreshold swing, multiscale simulations

## Abstract

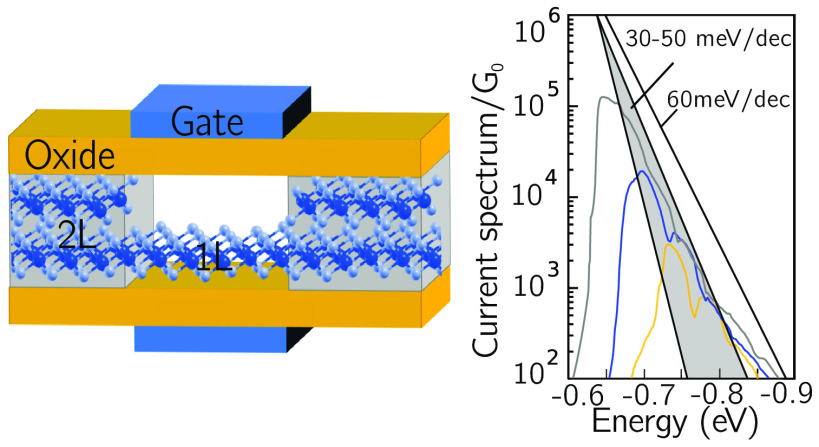

The bandgap dependence on the number of atomic layers of some families of two-dimensional (2D) materials can be exploited to engineer and use lateral heterostructures (LHs) as high-performance field-effect transistors (FETs). This option can provide very good lattice matching as well as high heterointerface quality. More importantly, this bandgap modulation with layer stacking can give rise to steep transitions in the density of states (DOS) of the 2D material that can eventually be used to achieve sub-60 mV/decade subthreshold swing in LH-FETs thanks to an energy-filtering source. We have observed this effect in the case of a PdS_2_ LH-FET due to the particular DOS of its bilayer configuration. Our results are based on *ab initio* and multiscale materials and device modeling and incite the exploration of the 2D-material design space in order to find more abrupt DOS transitions and better suitable candidates.

Semiconductor heterostructures of the III–V and II–VI materials systems have played a fundamental role in the progress of electronics and optoelectronics. First proposed by Kroemer in the 1950s,^1^ they have been involved in the invention of quantum-well lasers^2^ and high-electron-mobility transistors.^3^ The large number of available two-dimensional (2D) materials and the possibility to combine them even in the presence of significant lattice mismatch has led to a new wave of interest in materials engineering based on heterostructures of 2D materials. In particular, 2D materials enable the realization of vertical heterostructures, also called “van der Waals” heterostructures, consisting in the vertical stacking of layers of different 2D materials loosely coupled by van der Waals interactions,^4,5^ and of lateral heterostructures (LHs), in which a single 2D layer consists of juxtaposed regions of different lattice-matched 2D materials.^6−9^

LHs have been shown to be particularly well suited as channel materials in high-performance field-effect transistors (FETs) for digital electronics.^10^ However, the quality of the heterojunction is one of the major obstacles toward the experimental demonstration of high-performance LH-FETs. The possibility of fabricating LHs by modulating the stacking order of a single 2D material provides the opportunity of perfect lattice matching and growth compatibility and therefore a chance to obtain high material quality.^11^

Recently, a particular group of transition-metal dichalcogenides (TMDs) involving noble transition metals (Pt, Pd, and Ni), combined with S, Se, and Te, have been predicted^12^ and demonstrated to have strong gap dependence on the number of stacked layers.^13^ The so-called “noble TMDs” are, thus, promising contenders to build 2D LHs by modulation of the number of layers of adjacent regions of the same material. Indeed, these structures based on noble TMDs, which strictly speaking could be considered homostructures instead of heterostructures, would be easier to realize than those made of different 2D materials. *Ab initio* calculations predict that the bandgap is reduced by more than 1 eV when these noble TMDs vary from monolayer (1L) to bilayer (2L), leading in some cases to a change of electronic phase from semiconductor to metal.^12,14^ Monolayer and few-layer PtS_2_ and PtSe_2_ have already been synthesized,^15−17^ and devices such as Schottky barrier diodes on silicon have been fabricated.^18^ More recently, FETs made of few-layer PdSe_2_ and PtSe_2_ have been experimentally realized showing ambipolar transfer characteristics,^13,19,20^ and a large dependence of PtSe_2_ conductance on the number of layers has been observed.^13^

There is an additional advantage of some noble TMDs in their bilayer form: Their density of states (DOS) exhibits a steep nonmonotonic variation around the Fermi energy that can be used as an energy filtering mechanism in order to obtain a subthreshold swing (SS) of the FET smaller than the Boltzmann limit of 60 mV/decade at room temperature. Indeed, in thermionic FETs, the inverse of the maximum slope of the current–voltage characteristics is limited to SS = *k*_B_*T*/*q* ln(10) per decade, where *k*_B_ is Boltzmann constant, *q* is the elementary charge, and *T* is the absolute temperature.^21−23^ This provides a room-temperature SS value of 60 mV/decade. A lower value can only be obtained if (1) injection is energy constrained, such as in a Tunnel FET^24,25^ or using impact ionization^26^ or (2) if an effective negative capacitance is realized in the gate oxide stack, thus amplifying the surface potential at the channel.^27,28^ In particular, the energy-constrained injection from a steep DOS source has been demonstrated very recently using a graphene source in combination with a CNT channel in ref (21) or a MoS_2_ channel in refs (22 and 23). We show here that this effect can be achieved in a system based on a single 2D material, specifically PdS_2_, where a bilayer source (injecting into a monolayer channel) can provide sufficient energy filtering to yield an SS below the Boltzmann limit.

In this work we investigate the potential of noble TMDs as channel materials for LH-FETs, showing the possibility to engineer an energy-filtering source in order to obtain sub-60 mV/dec SS at room temperature and to design devices with competitive figures of merit when compared to the predictions of the International Roadmap for Devices and Systems (IRDS).^29^ Finally, we show that noble TMDs enable the realization of a 2D resonant tunneling diode (RTD) based on LHs obtained with modulation of the number of atomic layers.

## Results and discussion

### Lateral Heterostructure FETs

We have selected those noble TMDs exhibiting a sharp nonmonotonic DOS around the Fermi level and therefore potentially able to achieve SS < 60 mV/decade at room temperature. Following bandstructure calculations available in the literature,^11,14^ we have opted for PdS_2_ and NiS_2_. Both materials are semimetal in bilayer form and semiconductors in monolayer form, so it is possible to build a FET with a lateral heterostructure formed by a bilayer source, a monolayer central channel region, and a bilayer drain. This results in two semimetal–semiconductor Schottky barriers at the source and drain ends. For the sake of clarity, a semimetal is a material with zero gap but a relatively low DOS around the Fermi energy. We have also considered PtS_2_ which presents a strong modulation of the bandgap from monolayer to bilayer, still maintaining a semiconducting gap.

**Figure 1:**
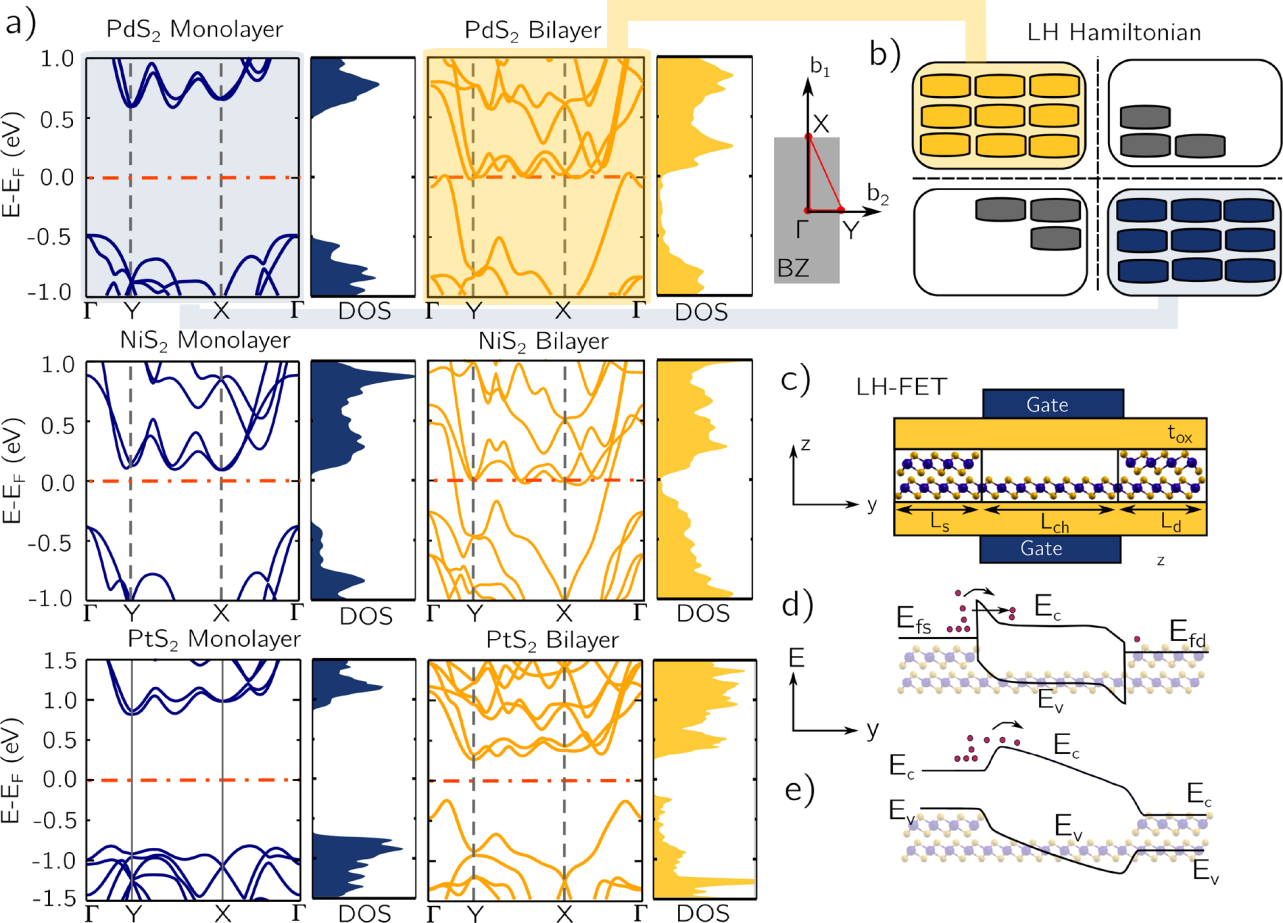
(a) Electronic band structure on a highly symmetric path along the Brillouin zone (depicted aside in gray with the path marked in red), and DOS integrated in the whole Brillouin zone, as computed with DFT, for monolayer (1L) and bilayer (2L) PdS_2_, NiS_2_, and PtS_2_. (b) Schematic representation of the construction of the lateral heterostructure Hamiltonian from the Hamiltonian of the bilayer and monolayer materials. (c) Schematic of the LH-FET, with bilayer source, monolayer channel, and bilayer-drain. (d) Illustration of the energy band edge profile of the PdS_2_ and NiS_2_ LH-FETs and (e) band edge profile of PtS_2_ LH-FET.

We have adopted a multiscale simulation approach combining different levels of physical abstraction, ranging from *ab initio* calculations of materials properties to full device simulations based on coherent quantum transport.^30^ We have calculated the electronic band-structure of monolayer and bilayer PdS_2_, PtS_2_, and NiS_2_ using density functional theory (DFT) as implemented in the Quantum Espresso suite^31^ (see Methods). The strong dependence of the electronic structure on the number of layers is highlighted in Figure 1a: When the crystal structure is varied from monolayer to bilayer, PdS_2_ and NiS_2_ undergo a phase change from semiconductor to semimetal, whereas PtS_2_ has its energy gap reduced from 1.59 to 0.48 eV. The drastic variation of the DOS of bilayer PdS_2_ and NiS_2_ around the Fermi level can be exploited to inject carriers from the source with submaxwellian energy tails, leading to SS < 60 mV/decade at room temperature.

The plane-wave DFT basis set has been translated into a maximally localized Wannier functions (MLWFs) basis set by means of the Wannier90 code,^32^ which provides us with tight-binding (TB) Hamiltonians for every material and stacking (see Methods and Figure S1 in the Supporting Information). The TB Hamiltonians are then employed to build a total Hamiltonian of the lateral heterostructure devices, following the procedure developed and validated in ref (33) (Figure 1b and Methods). In order to accurately model the Schottky barrier formed at the bilayer/monolayer interfaces, we have performed an energy analysis from first-principles taking into account the band offsets and the formation of dipoles^34^ (see Supporting Information).

The considered LH-FETs are also illustrated in Figure 1c. The length of the bilayer source and drain regions are *L*_s/d_ = 11, 10.4, and 16.7 nm for PdS_2_, NiS_2_, and PtS_2_ respectively. They are assumed to be ohmically contacted by the external metal leads with work functions of 5.6 and 5.8 eV for PdS_2_ and NiS_2_, respectively, and 5.3 eV/6.0 eV for the n-type/p-type PtS_2_. The monolayer 2D channel, with length *L*_ch_, is embedded in top and bottom SiO_2_, with thickness *t*_ox_ = 0.5 nm.

Sketches of the band-edge profiles of the PdS_2_ and NiS_2_ LH-FETs with semimetal source and drain are shown in Figure 1d; the PtS_2_ LH-FET with small gap source and drain is shown in Figure 1e. The complete MLWF Hamiltonian describing the channel including source and drain regions feeds the open-boundary Schrödinger equation, within the non-equilibrium Green’s functions (NEGF)^35^ formalism, that is self-consistently solved with the electrostatics of the whole device (see Methods).^36,37^

**Figure 2:**
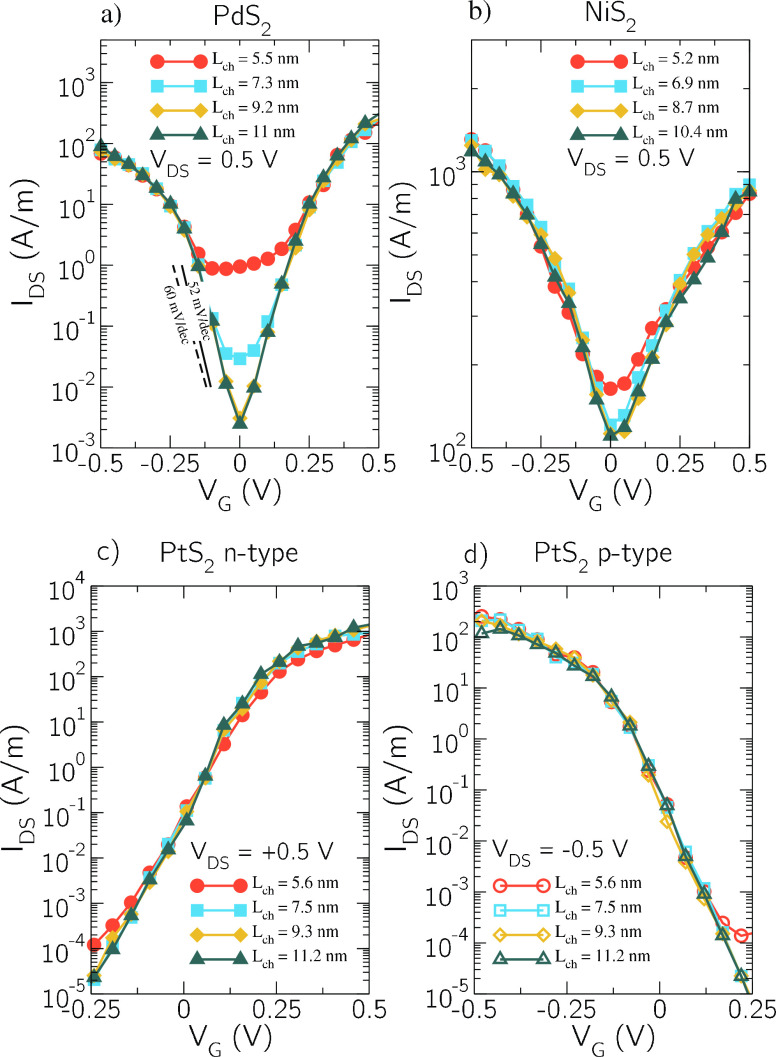
Transfer characteristic in semilogarithmic scale for the LH-FETs with PdS_2_ (a), NiS_2_ (b), and p-type/n-type PtS_2_ (c)/(d) considering a channel length ranging from ≃5 nm up to ≃10 nm and drain-to-source voltage |*V*_DS_| = 0.5 V. In the case of PdS_2_ and NiS_2_, an ambipolar behavior is observed due to the semimetallic source and drain. In the case of PtS_2_, we consider both the nFET and the pFET. The channel is always undoped. A metal gate workfunction of 5.52 eV is assumed in the PdS_2_ and NiS_2_ devices to observe the crossover between n-type and p-type conduction at *V*_GS_ = 0 V. For the PtS_2_ nFET and pFET, the gate workfunction is tuned to 5.28 and 6.2 eV, respectively, to set *I*_off_ = 100 nA/μm at *V*_GS_ = 0 V.

In order to study the potential performance of the considered LH-FETs for logic applications, we set a supply voltage *V*_dd_ = 0.5 V, and we simulate the transfer characteristics for a drain-to-source voltage *V*_DS_ = *V*_dd_. Those are shown in Figure 2 in semilogarithmic scale for channel lengths ranging from *L*_ch_ ≃ 5 nm up to *L*_ch_ ≃ 10 nm and for the three different materials. As it can be seen, the bilayer metallic source/drain regions in PdS_2_ and NiS_2_ lead to an ambipolar behavior. For channel lengths longer than 10 nm, *I*_off_ is not further reduced, being the ambipolarity determined by the bandgap of the monolayer channel and the capability of the bilayer source to inject both kinds of carriers. In this regard, some improvement might be achieved following the back-gate over/underlapping strategy at the source/drain discussed in ref (38). Here we have tuned the gate workfunction in the PdS_2_ and NiS_2_ devices to 5.52 eV so to observe the crossover between n-type and p-type conduction at *V*_GS_ = 0 V.

The PtS_2_ LH-FET is not ambipolar since it has semiconductor source and drain. In this case we show in Figure 2c,d both the nFET and the pFET characteristics, where the gate workfunction is tuned to 5.28 and 6.2 eV, respectively, to obtain the current in the OFF state *I*_off_ = 100 nA/μm at *V*_GS_ = 0 V, as required by the IRDS^29^ for high-performance (HP) applications where the OFF state corresponds to *V*_GS_ = 0 V and *V*_DS_= ±*V*_dd_. The source and drain regions have a donor doping with molar fraction *N*_D_ = 4.1 × 10^–2^ in the case of nFET and acceptor molar fraction *N*_A_ = 9.5 × 10^–2^ in the case of pFET. The channel is undoped for all devices.

**Figure 3:**
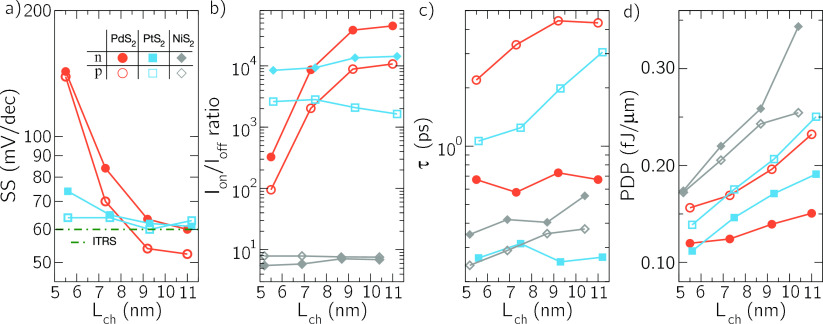
SS, *I*_on_/*I*_off_, τ, and PDP as a function of the channel length, *L*_ch_, for the LH-FETs based on PdS_2_, PtS_2_, and NiS_2_, evaluated according to the IRDS requirements for high-performance applications: *I*_off_ = 100 nA/μm.

Interestingly, the p-type branch of the PdS_2_ LH-FET *I*_DS_–*V*_GS_ curve exhibits a submaxwellian SS down to 52 mV/decade, as shown in Figure 3a. The possibility to achieve sub-60 mV/decade SS in a thermionic FET has been a subject of debate.^39,40^ In particular, in ref (40) the authors argue that the 60 mV/decade limit cannot be beaten in a single barrier device, although they eventually conclude that the role of the DOS can be essential to reverse this situation, as has already been experimentally demonstrated in the case of a graphene source.^21^

**Figure 4:**
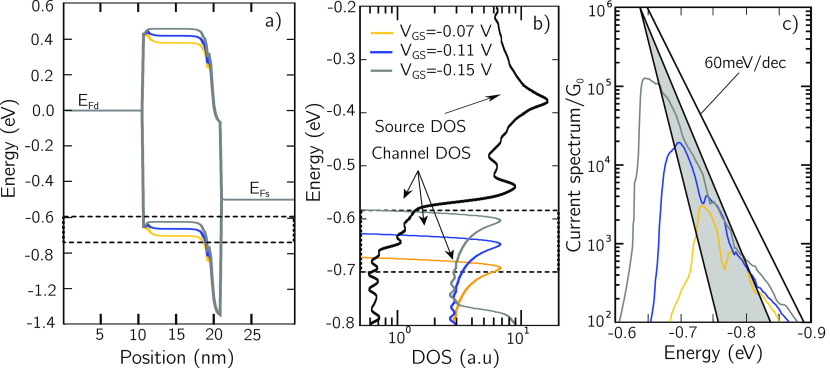
(a) Conduction and valence band edge profiles of the PdS_2_ LH-FET for *V*_DS_ = −*V*_dd_ and three different values of *V*_GS_ = −0.07, −0.11, and −0.15 V in the subthreshold region of the p-branch. (b) DOS of the source and of the channel for the corresponding values of *V*_GS_ obtained in a dense *k*-mesh grid, see Supporting Information, and after a Gaussian smoothing for the energy integration with σ = 10 meV. (c) Current spectrum as a function of energy. The range of slopes between 30 mV/decade and 50 mV/decade is in the shaded gray. The 60 mV/decade Boltzmann limit is also plotted.

We can discuss the specific mechanism in the PdS_2_ LH-FET by considering the band edge profiles shown in Figure 4a for *V*_DS_ = *V*_dd_ and for three different values of *V*_GS_ in the subthreshold region of the p-type branch. The Schottky barriers of drain–channel and source–channel junctions are calculated from first principles (see Supporting Information). A variation of *V*_GS_ modulates both the conduction and valence band edges in the channel and the transparency of the Schottky barrier between source and channel. The drop of the DOS in the source for energy below the source Fermi level (corresponding to −0.5 eV for the considered bias point) is clearly visible in Figure 4b, where the DOS is obtained from refined calculations applying, for visualization purposes, a Gaussian smoothing with σ = 10 meV (see Supporting Information), and is responsible for a sharper energy filtering than that provided by the Maxwell–Boltzmann tail of the occupation factor, and therefore for an SS lower than the so-called Boltzmann limit. This is even more apparent by considering the energy spectrum of the current, that is, the current density per unit energy, for different values of *V*_GS_ shown in Figure 4c. The slope of the logarithm of the current spectrum as a function of energy would be exactly −1/(*k*_B_*T*) for a constant DOS, corresponding to one decade every 60 meV at room temperature. Due to the nonconstant DOS in the source, such a slope is however not constant, and in one decade around an energy of −0.7 eV, where the DOS is dramatically quenched, it is in the range of 30–50 meV, marked as a shaded region in Figure 4c. This more confined current spectrum is responsible for the sub-Maxwellian subthreshold swing of the p-type branch transfer characteristics of the PdS_2_ LH-FET.

Energy filtering is not observed in the n-branch because the DOS of the conduction band states in PdS_2_ do not show a similar steep transition (see Supporting Information for more details). The effect is not observed in NiS_2_ where the small gap of the monolayer semiconductor results in large interband tunneling currents and very poor SS (above 200 meV/decade and therefore not shown in Figure 3a). The PtS_2_ LH-FET has a close to ideal Maxwellian SS of 60 mV/decade for HP applications due to the dominant Fermi window tail that restricts the SS.

Other transistor figures of merit for digital electronics are considered in Figure 3 following the IRDS specifications for HP applications. In particular, Figure 3b shows *I*_on_/*I*_off_ ratio, where *I*_on_ is the drain current in the ON state, corresponding to *V*_GS_ = *V*_DS_ = ±*V*_dd_ and *I*_off_ = 100 nA/μm, is the current in the OFF state as defined by the IRDS for HP. To this purpose, the PdS_2_ LH-FET exhibits the highest *I*_on_/*I*_off_ ratio for channel length close to 10 nm thanks to the lower SS just discussed, but not for shorter channel lengths (down to 5 nm), because of the high *I*_off_ due to ambipolarity and large source-to-drain tunneling. The situation is worse for the NiS_2_ LH-FET: Its smaller monolayer bandgap (0.47 eV) leads to a very poor *I*_on_/*I*_off_ ratio. The PtS_2_ LH-FET, instead, has a semiconducting bandgap and negligible source-to-drain tunneling and therefore exhibits high *I*_on_/*I*_off_ ratio for *L*_ch_ down to 5 nm.

Relevant figures of merit for transistor performance in digital circuits are also the intrinsic delay time τ = (*Q*_on_ – *Q*_off_)/*I*_on_ and the power delay product (PDP) = *V*_dd_*τI*_on_, where *Q*_on_ and *Q*_off_ are the total mobile charge in the channel in the ON and OFF states, respectively (Figure 3c,d). The nFETs based on PdS_2_ and PtS_2_ exhibit expected τ and PDP compliant with IRDS requirements for next technology nodes,^10,29^ together with an *I*_on_/*I*_off_ ratio close to 10^4^ for channel lengths of at least 10 nm, which implies acceptable stand-by power consumption for HP applications.^29^ The pFETs have slightly worse PDP and τ than the nFETs, due to the smaller source DOS in the valence band, apparent in Figure 1a, which is responsible for a smaller *I*_on_, as can be seen in the asymmetric transfer characteristics of Figure 2.

While the ambipolarity of PdS_2_ and NiS_2_ FETs spoils their use in low-power (LP) applications, PtS_2_ FETs also satisfy the IRDS requirements to this purpose (*i*.*e*., they reach an *I*_off_ = 100 pA/μm) achieving *I*_on_/*I*_off_ ratios above 10^6^ and 10^5^ for n-type and p-type FETs, respectively (see Supporting Information). For low stand-by power, SS is spoiled in shorter channel lengths since the tunneling current becomes comparable to the pursued *I*_off_: For *L*_ch_ ≈ 5–8 nm it is in the range 80–100 mV/decade for nFETs and 70–90 mV/decade for pFETs, deviating considerably from the 60 mV/decade observed in longer channels. Finally, the PDP is slightly lower and the intrinsic delay time slightly higher than the values obtained for HP (see Supporting Information for details).

### Resonant Tunneling Diode

**Figure 5:**
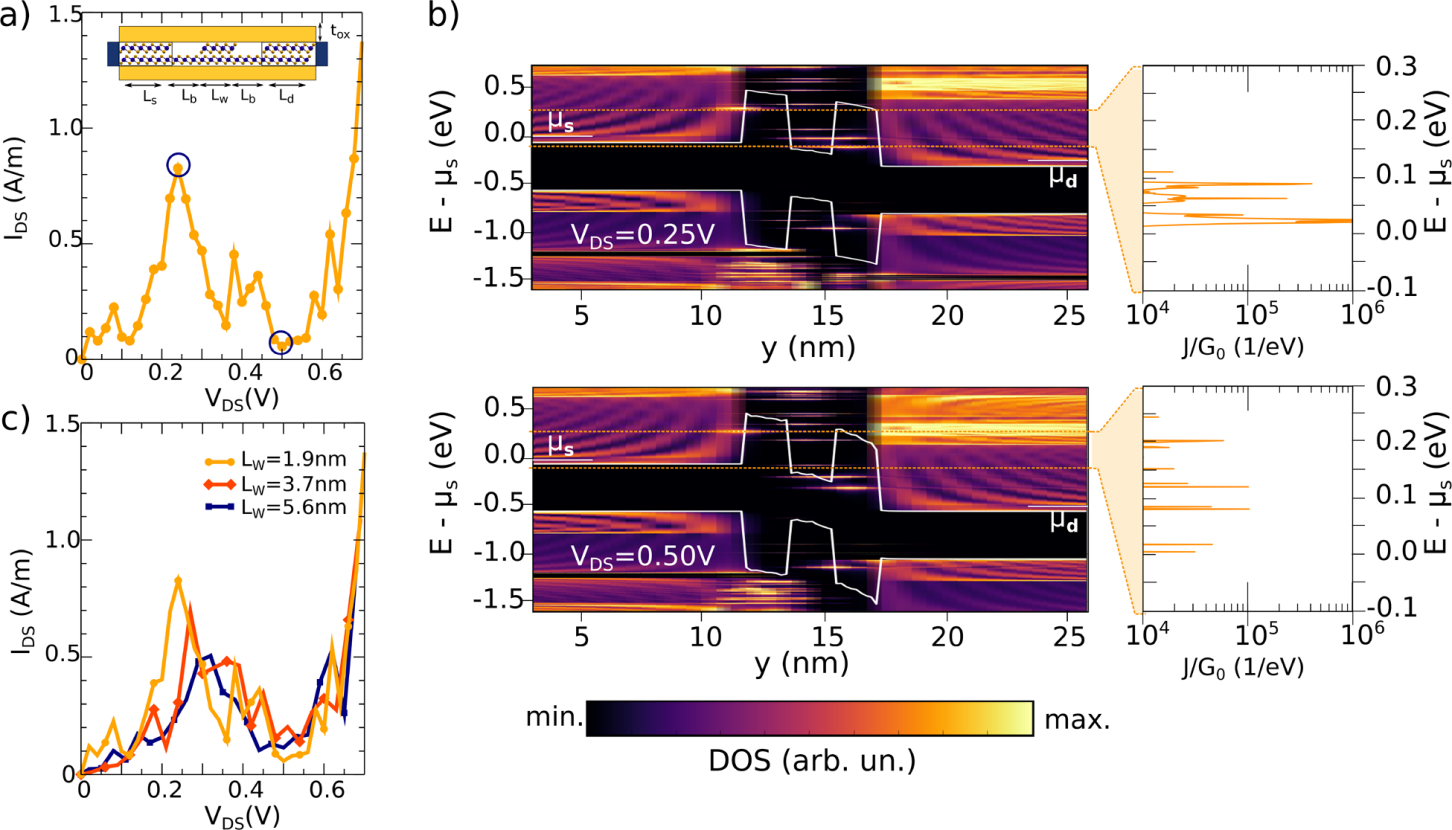
(a) *I*_DS_*vsV*_DS_ characteristic of the LH-RTD based on PtS_2_ with *L*_w_ = 1.9 nm. Inset: Schematic of the LH-RTD. (b) Local DOS as a function of the energy and the position along the device length and current-density spectrum, normalized to the conductance quantum (*G*_0_ = 2*q*^2^/*ℏ*), for the RTD with a *L*_w_ = 1.9 nm, and for *V*_DS_ = 0.25 and 0.5 V. (c) Current–voltage characteristics of the LH-RTD for different well lengths.

Finally, we have studied the operation of a 2D resonant tunneling diode based on a PtS_2_ LH (LH-RTD). We have considered bilayer PtS_2_ drain and source regions with a length of 11.2 nm. Two monolayer regions act as energy barriers confining a bilayer well. The barrier length is *L*_b_ = 1.9 nm, and the length of the well *L*_w_ is varied from 1.9 nm up to 5.6 nm. The whole 2D channel is embedded in SiO_2_. Figure 5a shows the *I*_DS_*vsV*_DS_ characteristics for *L*_w_ = 1.9 nm at room temperature. The current has a clear nonmonotonic behavior exhibiting a pronounced negative differential resistance, due to resonant tunneling through quantized states in the 2D well. The local DOS in the channel is plotted in Figure 5b as a function of energy and of position along the device length (*y*) for *V*_DS_ = 0.25 and 0.5 V, corresponding to the main peak and to the valley of the current–voltage characteristics. Let us stress the fact that the bandstructure of different regions is fully considered in the calculation, but inelastic processes and heterojunction defects are not included.

The Fermi level at the source (μ_s_) and drain (μ_d_) leads are marked. We have superimposed the conduction and valence band profiles to the local DOS colormaps. The alignment of μ_s_ with these quantized energy levels results in resonances in the current spectrum density and is controlled by *V*_DS_. We have also explored different well lengths: *L*_w_ = 1.9, 3.7, and 5.6 nm (Figure 5c), observing that the NDR effect is preserved, although the position of the peak varies with the well length as so does the energy quantization, while the position of the valley is not modified as it depends on the height of the barrier limiting the thermionic emission.

## Conclusion

We have shown that the strong dependence of the bandgap upon the number of layers of noble TMDs can be used to devise electron devices based on transport through lateral heterostructures of TMDs, such as LH-FETs and LH-RTDs. We have used *ab initio* multiscale simulations to demonstrate that LH-FETs based on PdS_2_ and PtS_2_ can comply with IRDS performance requirements^29^ for future integrated circuit technology for high-performance digital applications. On the other hand, LH-FETs made of NiS_2_ cannot meet such requirements, due to the low gap and ambipolar behavior.

We have also predicted the steep (submaxwellian) subthreshold behavior of p-type FETs based on PdS, due to the asymmetry of the bilayer PdS_2_ DOS around the Fermi level, achieving SS = 52 mV/decade. The demonstration of this effect in an intrinsic 2D material observed here can be further exploited for device design. It must be noted that in the presence of electron–electron or electron–phonon scattering, the behavior of the studied devices would be degraded with respect to the optimum ballistic condition assumed here, with direct impact on the achieved sub-Boltzmann slope. Moreover, the SS value for PdS_2_ FETs is not expected to boost the device performance stunningly as compared to the conventional limit. However, and more importantly, the results presented here confirm that the 60 mV/decade limit can be beaten in 2D-based FETs, as it has been proved experimentally in graphene, encouraging the exploration of new 2D materials with sharper DOS and consequently steeper SSs.

Finally, we have also predicted the possibility of using 2D LHs to obtain a resonant tunneling diode, with a pronounced peak-to-valley ratio of the current–voltage characteristics, which is suitable for experimental observation.

## Methods

Density functional theory as implemented in Quantum Espresso code has been employed to determine the electronic structure of PdS_2_, NiS_2_, and PtS_2_. The crystal geometry of monolayer and bilayer 2D crystals is characterized by a 1T arrangement, with a layer of Pt/Pd/Ni atoms sandwiched between two atomic layers of S atoms. The atom coordinates and lattice vectors have been obtained after,^14^ where a structural optimization of the unitary cell was performed. We have considered 40 Å of vacuum in the direction orthogonal to the 2D layers to minimize spurious interactions between periodic repetitions of the cell. For the exchange–correlation functional, the local density approximation has been considered under the Perdew–Zunger^41^ parametrization within norm-conserving pseudopotentials. The energy cutoffs for charge density and wave function expansions have been set to 360 and 60 Ry, respectively. A Monkhorst–Pack 10 × 5 × 1 *k*-mesh has been used for the Brillouin-zone integration, and an energy convergence threshold of 10^–6^ eV in the iterative solution of the Kohn–Sham equations was ensured. Additionally, an analysis of the Schottky barriers formed at the 2L/1L heterojunction has been performed (see Supporting Information).

Maximally localized Wannier functions (MLWFs) have been obtained by means of the Wannier90 code^32^ for every material and stacking. For the change of basis, the same *k*-sampling of the Brillouin zone as in the DFT simulations has been used to compute the overlap matrices required to determine the MLWFs. Twelve bands around the fundamental gap have been considered, and a threshold of 10^–10^ Å has been set for the total spread change in the MLWFs in 20 consecutive iterations. The MLWFs band structures have been calculated along the same path as in DFT, showing very good agreement (see Supporting Information Figure S1). The MLWF Hamiltonians of the 1L and 2L regions have been employed to build the total Hamiltonian of the lateral heterostructure following the procedure presented in ref (33).

The device simulations consist of the self-consistent solution of the open-boundary Schrödinger equation, within the non-equilibrium Green’s functions (NEGF)^35^ formalism, and the Poisson equation, for which we have used the open-source code NanoTCAD ViDES.^36,37^ The construction of the Hamiltonian of the heterostructure from the Hamiltonians of the different regions/materials requires a careful treatment, with special attention to the mixing of the interface elements. We have followed the procedure we developed and validated in ref (33). In particular, the off-diagonal elements connecting the 1L and 2L regions and determining their coupling have been assumed to be equal to those of the monolayer region. We have tested different alternatives for the off-diagonal coupling elements at the interface (see Supporting Information) observing little variation in the device behavior. This mixing procedure provides the best results in terms of robustness in the convergence, preserving computational accuracy as compared to *ab initio* simulations. For all devices we have considered operation at temperature of 300 K.

## Supporting Information Available

Additional details on the electronic band structure of monolayer and bilayer PtS_2_, PdS_2_, and NiS_2_ and on the Hamiltonian construction as well as the current spectrum for the PdS_2_ LH-FET (PDF)
