# Supplementary material for: Lateral Heterostructure Field-Effect Transistors Based on Two-Dimensional Material Stacks with Varying Thickness and Energy Filtering Source
Source: ACS Nano. 2020 Jan 14;14(2):1982–9. doi: 10.1021/acsnano.9b08489 (PMC7993756; doi:10.1021/acsnano.9b08489)
Supplement: Supplementary file 1 — nn9b08489_si_001.pdf [file nn9b08489_si_001.pdf]

Supplementary Information: Lateral Heterostructure Field-Effect Transistors Based on 2D-Material Stacks with Varying Thickness and Energy Filtering Source

Enrique G. Marin,[†,‡] Damiano Marian,[†] Marta Perucchini,[†] Gianluca Fiori,[†] and Giuseppe Iannaccone[*,†]

Dipartimento di Ingegneria dell'Informazione, Università di Pisa, 56122, Pisa, Italy, and Dpto. Electrónica, Fac. Ciencias, Universidad de Granada, 18071, Granada, Spain

E-mail: giuseppe.iannaccone@unipi.it

[*]To whom correspondence should be addressed
[†]Universita' di Pisa
[‡]Universidad de Granada

Hamiltonian of the lateral heterostructure

In order to build the heterostructure Hamiltonian, one must take notice of the off-diagonal elements connecting the monolayer and the bilayer regions. In particular, in the Hamiltonian along the transport direction each block represents a (#wannier centers × #wannier centers) matrix, and the number of blocks along a row is constrained by the Monkhorst-Pack grid. The blocks in the off-diagonal elements connect a row of the bilayer region to a column of the monolayer region, i.e. they connect the last cell of the source and the first of the channel (and equivalently at the drain end).

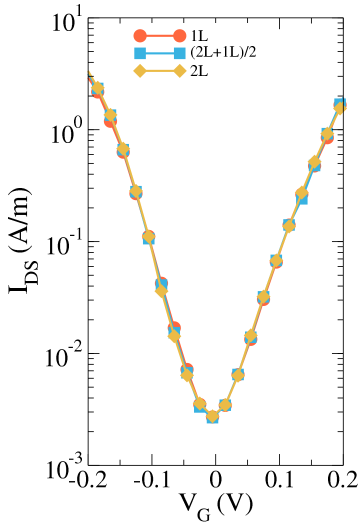

Figure 1: Transfer characteristic for the bilayer-monolayer-bilayer PdS_2 lateral-heterostructure FET with a channel length 9.2 nm assuming different coupling between the bilayer and monolayer regions.

We have tested how different couplings between the monolayer and bilayer affect to the device. Figure 1 shows the transfer response of the PdS_2 LH-FET with 9.2-nm long channel, comparing three mixing strategies: 1) the one employed in the manuscript, where the off diagonal elements are assumed to be equal to those of the monolayer region (red circles) 2) a mean of the 1L and 2L coupling values (blue squares), and 3) the bilayer coupling values (yellow diamonds). We observe that regardless the values assigned to the off-diagonal coupling parameters the transfer characteristic change very little and the SS<60mV/decade is conserved.

Band structure

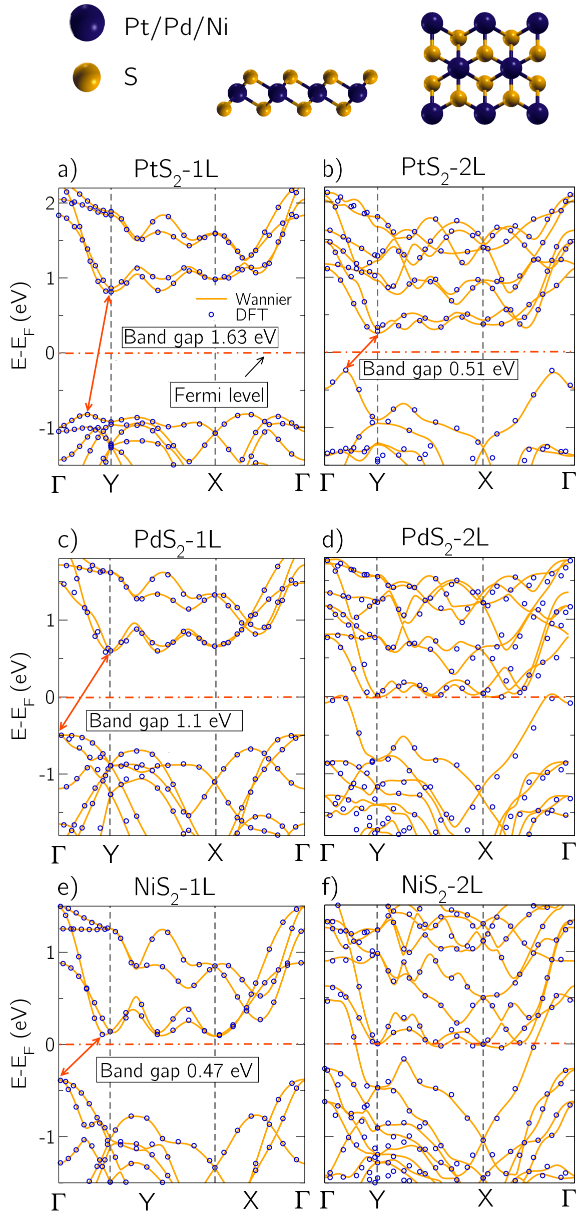

Figure 2: Top and lateral views of the 1T crystal structure of monolayer and bilayer PtS_2, PdS_2, and NiS_2. They are characterized by a 1T structure, with a layer of Pt/Pd/Ni atoms sandwiched between two atomic layers of S. Electronic band-structure on a highly symmetric path along the Brillouin zone as computed with Density Functional Theory calculations (symbols) and with Maximally Localized Wannier Functions (lines).

Schottky Barrier

To deepen in the analysis of the 2L-1L interface and obtain accurate information on the Schottky Barrier (SB) formation and the alignment of the bandstructures of the two regions, we have performed an analysis of a complete 2L-1L heterojunction. First principles calculations using the Quantum Espresso suite[1] (see Methods) have been performed for the 2L-1L heterojunction. The profile of the vacuum level and the potential profile with respect to the Fermi level, E_F, has been calculated taking into account the formation of dipoles but neglecting the presence of defects. Following the same methodology as in,[2] we have extracted values for the SBs of 0.43 eV, 0.26 eV and 0.41 eV for the PdS_2, NiS_2 and PtS_2 lateral heterostructures, respectively. These values have later been considered in the construction of the Hamiltonian for the device simulations.

Current spectrum for the *p*-type PdS_2 LH-FET

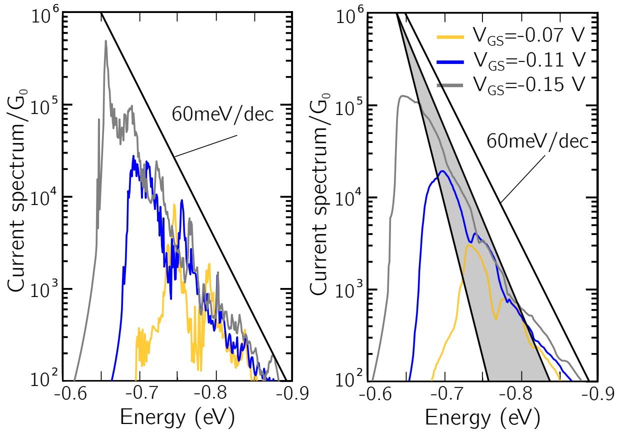

Figure 3: (Left) Current spectrum for the $L_{\mathrm{ch}} = 9.2$ nm PdS_2 LH-FET for the bias points in the p-branch where the sub-maxwellian SS is achieved. (Right) In order to appreaciate better the slope of the current spectrum vs. energy we low-pass filtered the current using an average energy window of 5 meV. In shaded gray the range of slopes between 30 mV/decade and 50 mV/decade. The 60 mV/decade Boltzmann limit is also plotted.

Current spectrum for the n-type PdS_2 LH-FET

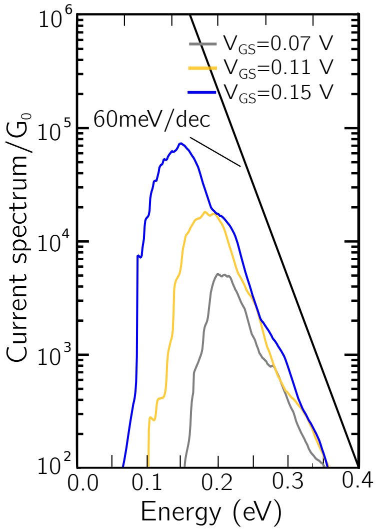

Figure 4: Current spectrum as a function of energy for the $L_{\mathrm{ch}} = 9.2$ nm PdS_2 LH-FET for three different values of $V_{\mathrm{GS}} = 0.07, 0.11, 0.15$ V in the subthreshold region of the n-type branch. The 60 mV/decade Boltzmann limit is also plotted. In order to appreaciate better the slope of the current spectrum vs. energy we low-pass filtered the spectrum using an average energy window of 5 meV.

Figures of merit of the PtS_2 LH-FET for low stand-by power applications

Table 1: Figures of merit for several channel lengths of the PtS_2 for low-power applications. Differently from Figure 3 in the main text (evaluated for high-perfomance applications) the IRDS low-power specifications sets $I_{\mathrm{off}} = 100$ pA/μm.

	n-type				*p*-type			
Ł$_{\mathrm{ch}}$ (nm)	SS (mV/dec)	I_{on}/I_{off}	τ (ps)	PDP (fJ/μm)	SS (mV/dec)	I_{on}/I_{off}	τ (ps)	PDP (fJ/μm)
5.6	108	$1.1 \cdot 10^6$	1.15	0.04	90	$3.4 \cdot 10^5$	6.1	0.10
7.5	80	$3.6 \cdot 10^6$	0.49	0.09	73	$7.5 \cdot 10^5$	3.4	0.13
9.3	70	$3.9 \cdot 10^6$	0.49	0.10	60	$7.5 \cdot 10^5$	3.8	0.14
11.2	63	$4.5 \cdot 10^6$	0.47	0.11	60	$6.3 \cdot 10^5$	4.8	0.15

Density of states calculation for the 1L and 2L PdS_2

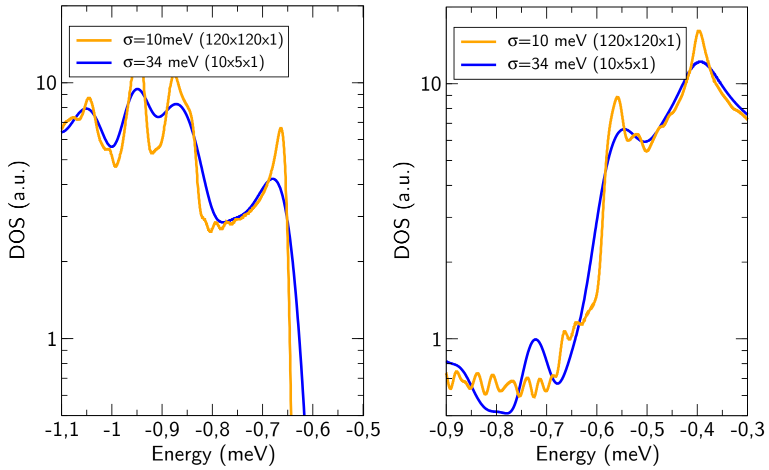

Figure 5: DOS of the monolayer (left) and bilayer (right) PdS_2 in a $10 \times 5 \times 1$ Monkhorst-Pack grid with 0.5 meV of energy resolution and considering a Gaussian smoothing for the energy integration $\sigma = 34$meV (blue) and in a $120 \times 120 \times 1$ Monkhorst-Pack grid with energy resolution 0.5 meV and after a Gaussian smoothing with $\sigma = 10$ meV (orange).

References

1. Giannozzi, P.; Baroni, S.; Bonini, N.; Calandra, M.; Car, R.; Cavazzoni, C.; Ceresoli, D.; Chiarotti, G. L.; Cococcioni, M.; Dabo, I.; Corso, A. D.; de Gironcoli, S.; Fabris, S.; Fratesi, G.; Gebauer, R.; Gerstmann, U.; Gougoussis, C.; Kokalj, A.; Lazzeri, M.; Martin-Samos, L. *et al.* QUANTUM ESPRESSO: A Modular and Open-Source Software Project for Quantum Simulations of Materials. *J. Phys.: Condens. Matter* **2009**, *21*, 395502.

2. Katagiri, Y.; Nakamura, T.; Ishii, A.; Ohata, C.; Hasegawa, M.; Katsumoto, S.; Cusati, T.; Fortunelli, A.; Iannaccone, G.; Fiori, G.; Roche, S.; Haruyama, J. Gate-Tunable Atomically Thin Lateral MoS_2 Schottky Junction Patterned by Electron Beam. *Nano Lett.* **2016**, *16*, 3788–3794.
